# Editorial: Inhibiting PARP as a Strategic Target in Cancer

**DOI:** 10.3389/fonc.2016.00091

**Published:** 2016-04-15

**Authors:** Kristin K. Zorn, Christina M. Annunziata

**Affiliations:** ^1^Department of Obstetrics and Gyencology and Divison of Genetics, University of Arkansas for Medical Sciences, Little Rock, AR, USA; ^2^National Cancer Institute, Bethesda, MD, USA

**Keywords:** PARP inhibitors, poly(ADP-ribose) polymerase, DNA repair, PARP-1, BRCA1 protein, cancer therapy

**The Editorial on the Research Topic**

**Inhibiting PARP as a Strategic Target in Cancer**

When Christina Annunziata and I embarked on guest editing an e-journal about poly(ADP-ribose) polymerase (PARP) inhibitors for cancer therapy, our goal was to capture how one of the most promising, rationally developed therapies had become increasingly complex in clinical use. We recruited an outstanding group of researchers to help in this effort. We organize their contributions into two broad categories, those with a more basic science approach and those with a more clinical approach, although these are not without overlap.

Beginning with the more mechanistic contributions, “The Elephant and the Blind Men: Making Sense of PARP Inhibitors in Homologous Recombination Deficient Tumor Cells” by De Lorenzo et al. provides an excellent review of the main models proposed to explain the synthetic lethality seen with PARP inhibition and deficiency in homologous recombination. Limitations of the models are highlighted, along with the potential impact that our knowledge gaps might have on clinical application of PARP inhibition. This article provides the groundwork for critical consideration of the other papers in this compilation.

In “Strategic Combination of DNA Damaging Agent and PARP Inhibitor Results in Enhanced Cytotoxicity,” Horton and Wilson describe using a mouse embryonic fibroblast cell culture model to better understand how PARP-1 inhibition impacts cell killing in combination with various chemotherapeutic agents. They report that the chemistry of the DNA repair intermediate that is formed is critical to PARP inhibitor-induced sensitization. This level of detail complements that presented by Steffen et al. who contributed “Structural Implications for Selective Targeting of PARPs.” The authors delve into the question of whether PARP inhibition should be targeted to all PARPs through binding to conserved regions or more selective in targeting specific PARPs. The answer has the potential to impact both PARP inhibitor effectiveness and toxicity.

Poly(ADP-ribose) polymerase enzymes are most widely recognized for their roles in single-strand DNA repair, especially when complementing the double-strand repair that is dependent on BRCA proteins. It is important to realize, however, that the PARP enzymes have other functions in the cell. In “Beyond DNA Repair: Additional Functions of PARP-1 in Cancer,” Weaver and Yang broaden our focus on PARP inhibition from the typically discussed DNA damage response to other processes, such as inflammation, angiogenesis, cellular metabolism, and cell death, that are critical to cancer biology. This information helps interpret some side effects of the PARP inhibitors in the clinic and hints at pathways to co-target in the future. In the group of more clinical contributions, Shah et al. build on this theme by discussing the clinical impact of our lack of complete understanding of the mechanism of action of PARP inhibitors in their contribution, “PARP Inhibitors in Cancer Therapy: Magic Bullets but Moving Targets.”

Key perspectives on the clinical development process of PARP inhibitor development unfold in additional articles included in the compilation. Ricks et al. discuss the development of PARP inhibitors from the perspective of the US Food and Drug Administration. In “Successes and Challenges of PARP Inhibitors in Cancer Therapy,” they provide insight into the regulatory aspects of the development process from the phase 0 veliparib trial to the current approval of olaparib for limited clinical use. Adding to this series, Burgess and Puhalla discuss clinical trials from their perspective as academic physicians. They have contributed an in-depth review of the use of PARP inhibitors in clinical trials to date in breast and ovarian cancer. Their review in “BRCA 1/2-Mutation Related and Sporadic Breast and Ovarian Cancers: More Alike than Different” provides a window into the biologic factors that might influence response to PARP inhibition. Additional detail is teased out, with relevance to women’s malignancies, in “The Role of PARP Inhibitors in the Treatment of Gynecologic Malignancies.” Here, Reinbolt and Hays review the data on the use of PARP inhibitors in ovarian, endometrial, and cervical cancer, and discuss their vision regarding future directions for their development. O’Sullivan et al. provide a complementary direction in “Beyond Breast and Ovarian Cancers: PARP Inhibitors for BRCA Mutation-Associated and BRCA-Like Solid Tumors.” Importantly, they broaden potential application with respect to tumor type, but focus on the molecular aspects that may help optimize PARP inhibitor use in a defined patient population. Specifically, these authors review the data for PARP inhibitor use in solid tumors other than breast and ovarian cancer, helping to frame the potential for expanded use in the future.

This collection of articles addresses the role of PARP inhibition in cancer therapy, from both basic science and clinical research perspectives. The integration of bench and bedside aspects is vital for moving the field forward to the most efficacious use of these agents. While our knowledge of PARP inhibitors has grown substantially in a relatively short amount of time, critical issues, such as mechanisms of action, appropriate therapeutic combinations, limiting short- and long-term toxicity, and defining the ideal patient population, remain to be resolved. We have compiled these articles to stimulate thoughts and discussion regarding this promising line of therapy, and expedite the successful application to patients.

## Author Contributions

All authors listed have made substantial, direct, and intellectual contribution to the work, and approved it for publication.

## Conflict of Interest Statement

The authors declare that the research was conducted in the absence of any commercial or financial relationships that could be construed as a potential conflict of interest.

